# Regulation of RNase PH during nutrient deprivation: the role of proteases, GroEL, and RNase II

**DOI:** 10.1128/mbio.00443-26

**Published:** 2026-04-07

**Authors:** Ashraf Hussain, Murray P. Deutscher

**Affiliations:** 1 Department of Biochemistry and Molecular Biology, University of Miami Miller School of Medicine 12235, Miami, Florida, USA; NYU Langone Health, New York, New York, USA

**Keywords:** exoribonuclease, Lon protease, *Escherichia coli*, protein degradation, regulation

## Abstract

RNase PH is a critical exoribonuclease in *Escherichia coli* that participates in tRNA maturation and RNA degradation. In earlier work, it was observed that levels of RNase PH decreased as much as 90% under conditions of nutrient deprivation, such as induced starvation and prolonged stationary phase, and that its removal was likely due to the degradation of the protein. Here, we examine the mechanisms involved in this regulatory process. We find that the protease Lon is primarily responsible for the removal of RNase PH that occurs in stationary phase and starvation. Conversely, RNase PH remains stable during the exponential phase of growth due to a protective interaction with the chaperonin protein, GroEL. Overproduction of GroEL protects RNase PH even under conditions of nutrient deprivation. Additionally, we find that RNase II activity also is required for the degradation of RNase PH, implying the involvement of an RNA molecule in the overall regulatory process. In mutant strains devoid of RNase II activity, even though retaining RNase II protein, RNase PH levels remain unchanged during nutrient deprivation which leads to excessive rRNA removal and ultimately to loss of viability. These findings provide another example of the complex regulatory mechanisms that underscore the importance of maintaining appropriate RNase levels under varying physiological conditions.

**IMPORTANCE:**

This work provides important new information on the regulation of a bacterial ribonuclease as it responds to nutrient deprivation. We find that RNase PH levels decrease up to 90% in late stationary phase and upon carbon starvation. We show that the enzyme is degraded by a protease under the stress conditions but that it is protected during growth by interaction with another protein, the chaperonin GroEL. This interaction does not occur under the stress conditions rendering RNase PH susceptible to degradation by protease Lon. We also find that the activity of another ribonuclease, RNase II, plays a role in the process, and that in the absence of RNase II activity, RNase PH does not decrease in stationary phase leading to cell death due to ribosome degradation. These studies identify a new mechanism of ribonuclease regulation and emphasize the importance of this regulation for cellular homeostasis.

## INTRODUCTION

Bacteria often adapt to environmental stress through regulatory processes involving ribonucleases (RNases). Since RNases are crucial for determining intracellular RNA levels (1–4), overproduction or underproduction of RNases can be harmful, leading either to the degradation of important RNAs or to poor stress adaptation, respectively (5–7). Therefore, it has become clear that the regulation of RNase levels and activity is essential, especially for cells under stressful conditions (6, 8). In addition, ribosomes, the cell’s protein factories, which usually are stable in growing cells, often become sensitive to degradation during these stress conditions due to an increase in free subunits that are much more sensitive to ribonucleases than intact ribosomes (9–11).

RNase PH is a 3′–5′, phosphorolytic exoribonuclease that plays a role in tRNA maturation and in structured RNA degradation (12, 13). In *E. coli,* starvation triggers rRNA degradation, and this process requires RNase PH which initiates nucleotide removal from the 3′ end of 16S RNA, leading to total rRNA breakdown by RNase E (14, 15). Common laboratory-grown strains of *E. coli*, such as MG1655, often contain a deletion of the *rph* gene since this appears to be a common mutation generated under stress conditions (16–18), indicating that the absence of RNase PH provides a selective advantage under such conditions. A second exoribonuclease, RNase II, is the major hydrolytic exoribonuclease in *E. coli* extracts, contributing about 90% of the total degradative activity present (19, 20). Although RNase II is not an essential enzyme, its removal significantly impacts rRNA degradation, tripling rRNA breakdown during starvation in what appears to be a counterintuitive response (14).

An earlier paper from this laboratory provided a partial explanation for these findings (21). In wild-type cells, RNase PH levels decrease dramatically under conditions of nutrient deprivation, such as stationary phase or induced starvation. In contrast, in cells devoid of RNase II, this decrease does not occur. The reduction in RNase PH is not due to regulation at the transcriptional level, but rather appears to be a consequence of a change in RNase PH stability. In wild-type cells, the decrease in RNase PH during nutrient deprivation limits initiation of ribosome breakdown, thus maintaining cell survival. However, in cells containing a mutated RNase II, the level of RNase PH remains elevated, leading to excessive ribosome degradation and eventually to cell death. The study by Sulthana et al. (21) revealed a possible new regulatory pathway for RNase PH and helped to explain why the *rph* gene frequently is deleted in lab evolution experiments and why the lack of RNase PH provides an advantage under stress conditions (16–18). Despite this information, much was still unclear about the mechanism of RNase PH depletion, how this was triggered during nutrient deprivation, and how RNase II participated in the regulatory process.

In this study, we begin to answer these questions. We find that the reduction in the amount of RNase PH in stationary phase is due to its degradation by the Lon protease, with some added effect of HslUV, another ATP-dependent protease. In growing cells, on the other hand, RNase PH remains relatively stable because it forms a stabilizing complex with the chaperonin protein, GroEL. However, as GroEL levels decrease when cells enter stationary phase (22), RNase PH becomes increasingly susceptible to the action of the degradative proteases. Overexpression of GroEL counteracts the proteolytic degradation of RNase PH. Additionally, we find that the role of RNase II in the regulatory process is dependent on RNase II activity, rather than RNase II protein, implying the participation of an as yet unknown RNA molecule in the regulation of RNase PH. These findings provide new insights into a complex regulatory network that controls the amount of RNase PH under stress conditions.

## RESULTS

### RNase PH decreases markedly in stationary phase

In earlier work, our laboratory found that the level of RNase PH decreased as much as 90% under conditions of nutrient deprivation engendered by induced carbon starvation (21). We noted at the time that starvation, although convenient as a rapid experimental protocol, is not a perfect mimic of the nutrient deprivation that occurs as cells enter and continue in stationary phase because the physiological changes that normally occur under these natural conditions do not have time to play out when rapid starvation is imposed.

**Fig 1:**
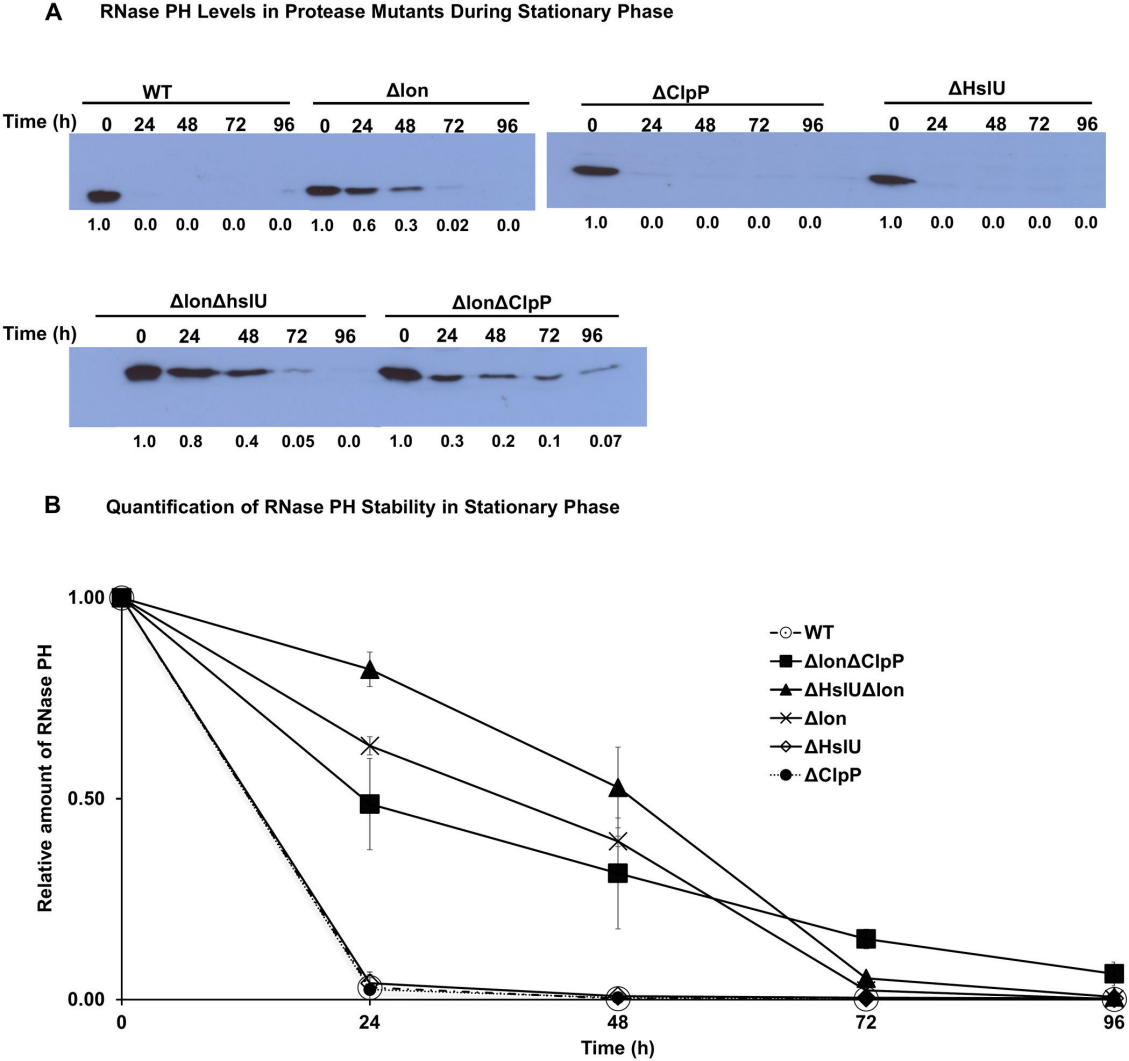
RNase PH stability in protease deletion mutants during stationary phase. (**A**) RNase PH levels in various protease mutants. Wild-type (WT) and protease deletion mutant strains (Δlon, ΔClpP, ΔHslU, ΔlonΔhslU, and ΔlonΔClpP) were grown to stationary phase in LB medium. Samples were collected at 0, 24, 48, 72, and 96 h and analyzed by Western blot using anti-RNase PH antibodies. Numbers below lanes represent relative band intensities quantified using ImageJ software, normalized to *T* = 0 h after background subtraction. Values represent mean ± SEM from *n* = 3 independent replicates. (**B**) Quantification of data from panel **A**.

For that reason, we sought to confirm for the experiments presented here that RNase PH levels also decrease markedly during stationary phase. As is shown in Fig. 1 and subsequent figures, RNase PH levels decrease, and the enzyme is essentially absent by 24 h of growth, largely mimicking what is seen after 3 h of starvation (21). Thus, nutrient deprivation occurring naturally as cells proceed deep into stationary phase or that induced by a starvation protocol are similar in many respects, and consequently, both methods will be used in the experiments presented here.

### Protease Lon is primarily responsible for RNase PH degradation during stationary phase and starvation

The pronounced reduction in RNase PH levels during starvation and prolonged stationary phase strongly suggested specific proteolytic degradation of the protein, and this was confirmed in earlier work (21). As a first step to understanding the mechanism of this regulation, it was important to define the proteases involved and to determine if they were pre-existing enzymes or newly activated ones that turned on in response to the nutrient deprivation.

To this end, single or double mutations of known proteases were introduced into strain MG1655*, and their effects on RNase PH levels during nutrient deprivation were determined. These proteases included Lon, ClpP, and HslU. As already noted, RNase PH disappears from cultures after 24 h of growth (Fig. 1), and this reduction in RNase PH levels is unaffected by the removal of either ClpP or HslU (Fig. 1). In contrast, the removal of Lon protease greatly stabilized RNase PH such that close to 70% of the protein was still present after 24 h and close to 40% remained after 48 h (Fig. 1). The removal of a second protease in addition to Lon generally had only a small additional effect (Fig. 1). Based on these findings, we conclude that Lon is the protease primarily responsible for the degradation of RNase PH in stationary phase. Nevertheless, it should be noted that RNase PH eventually disappears even in the absence of Lon protease suggesting that other proteases may also contribute to RNase PH degradation. Since there was some increased stabilization of RNase PH upon additional removal of ClpP or HslU, these proteases may play a role. We have been unable to generate a triple protease mutant to definitively answer this question.

**Fig 2:**
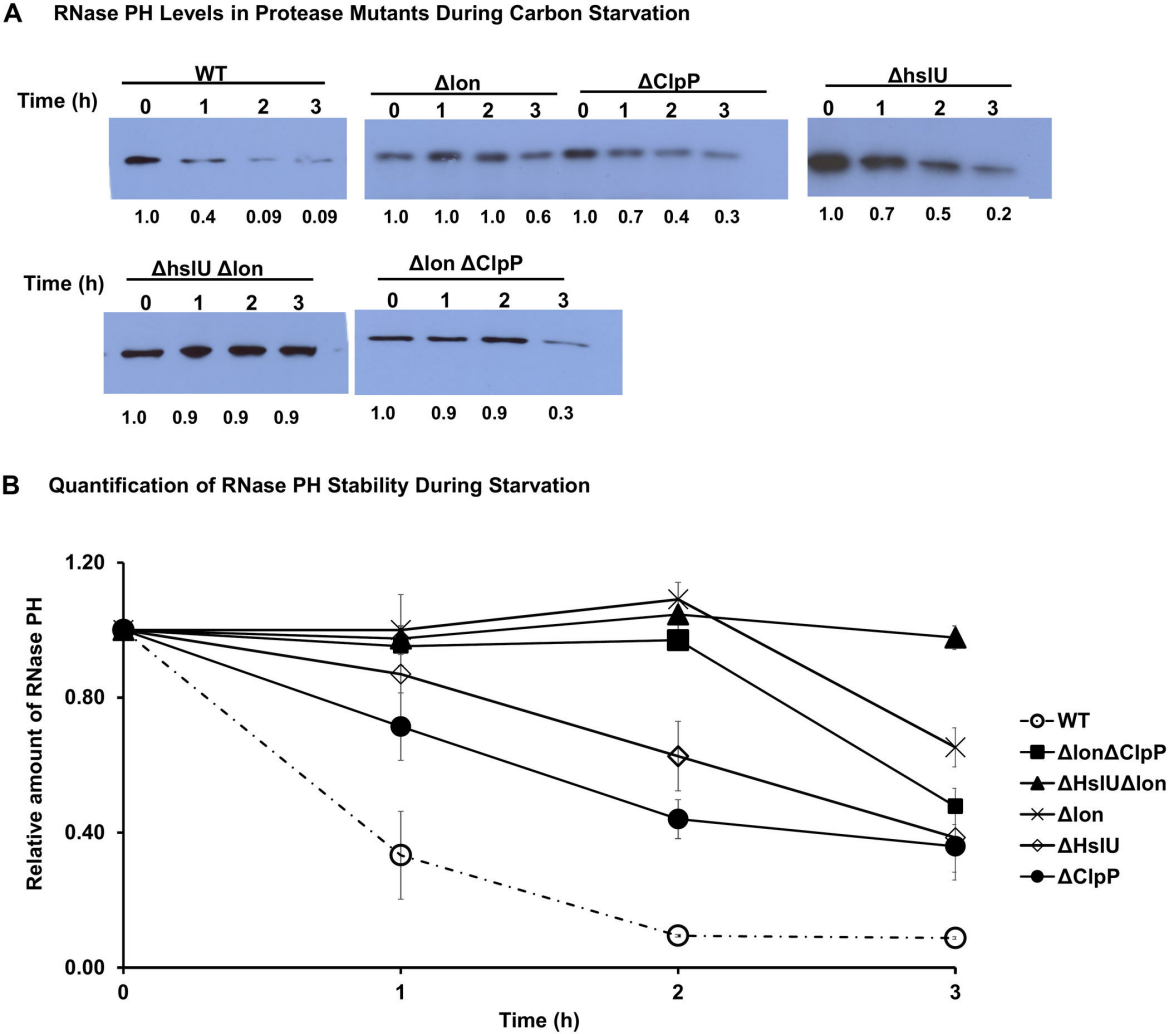
RNase PH stability in protease deletion mutants during carbon starvation. (**A**) RNase PH levels during glucose starvation. Wild-type and protease deletion mutant strains were subjected to carbon starvation by transfer to M9 minimal medium without glucose. Samples were collected at 0, 1, 2, and 3 h and analyzed by Western blot. Numbers below lanes represent relative band intensities quantified using ImageJ and normalized to *T* = 0. Values represent mean ± SEM from *n* = 3 independent replicates. (**B**) Quantification of data from panel **A**.

A similar situation was evident during starvation (Fig. 2). Again, Lon was the protease primarily responsible for the degradation of RNase PH, but in this instance, the removal of ClpP or HslU also led to significant stabilization of the RNase protein. In the absence of Lon, RNase PH was completely stabilized for 2 h, but some degradation occurred during the third hour of starvation. The removal of HslU as well as ClpP enhanced stability during the 3 h starvation period. These data indicate that during starvation, known proteases can account completely for the degradation of RNase PH under this condition.

### RNase PH forms a cross-linked complex during the exponential phase, but not during the starvation or stationary phase

While RNase PH is relatively stable during normal exponential growth (21), the data presented to this point show that it is proteolytically degraded under conditions of nutrient deprivation during stationary phase or induced starvation. Inasmuch as proteases are present during all these conditions, these findings suggest that some change in RNase PH structure likely occurs during nutrient deprivation that renders it sensitive to the action of proteases. Such a change, among multiple possibilities, could include the addition or removal of post-translational modifications or the presence or absence of an interaction with other macromolecules (see reference 8) for a detailed review of this topic). One post-translational modification already known to regulate RNase stability is acetylation. For example, acetylation of RNase R on a single lysine residue leads to its destabilization in exponential phase cells, whereas the absence of the modification in stationary phase cells results in a large increase of the RNase (23). Based on this prior example, we examined whether such a mechanism might be operating in the stabilization of RNase PH in exponential phase cells described here. However, using antibody directed against N-acetyl lysine, we were unable to detect this modification on RNase PH under any growth condition (Fig. S1), suggesting that acetylation does not play a role in any structural change envisioned for this RNase.

Second, we examined whether RNase PH might be associated with another macromolecule under any of the growth conditions studied. For this purpose, exponentially growing cells, starved cells, or cells in stationary phase were treated with varying concentrations of formaldehyde to generate crosslinks between RNase PH and any protein or RNA molecule that might be associated with it. After crosslinking, samples were then analyzed by gel electrophoresis using antibody directed against the FLAG-tag placed on RNase PH. Some samples also were treated with RNase A prior to electrophoresis to test for crosslinking to RNA.

**Fig 3:**
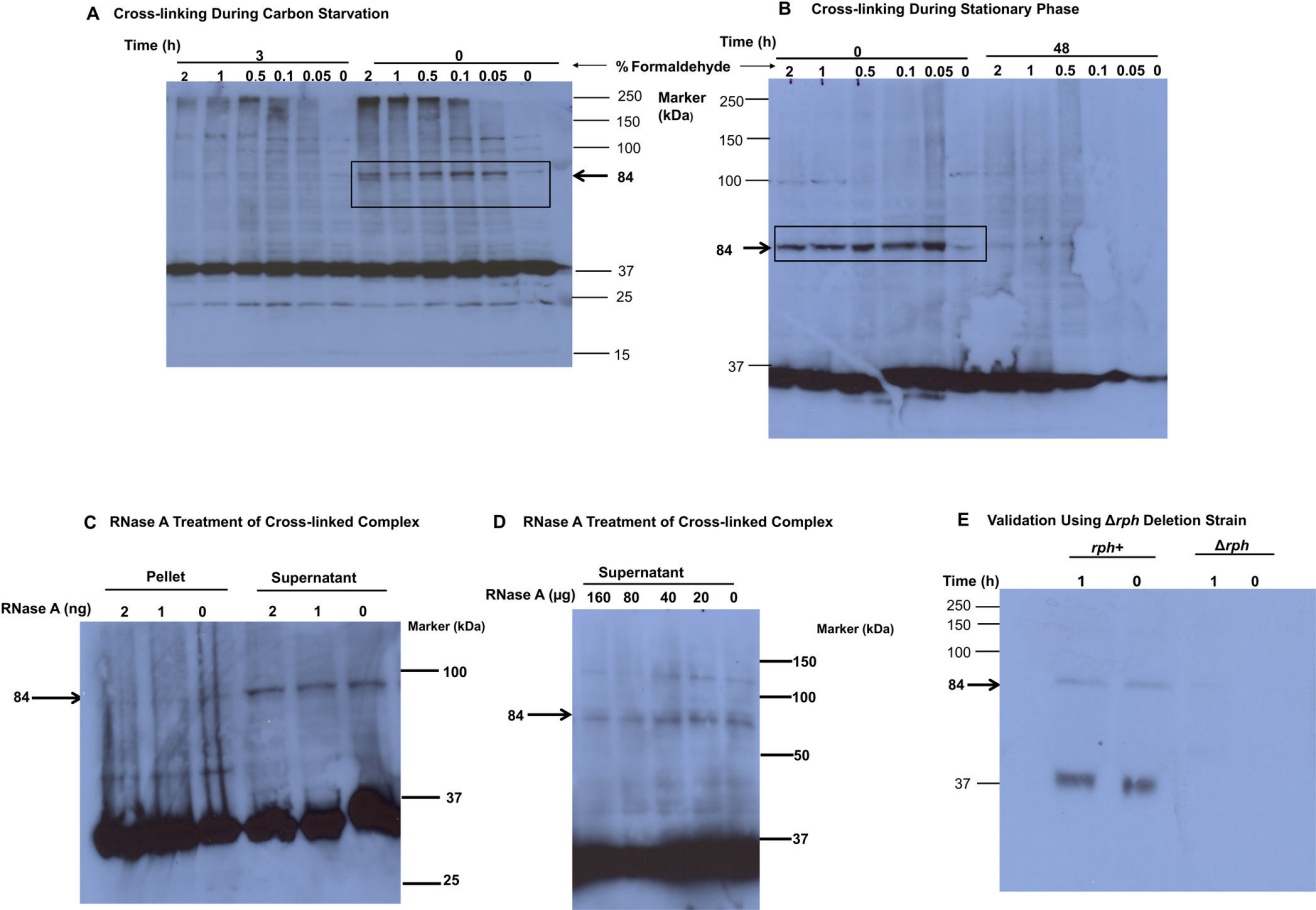
RNase PH crosslinking during starvation and stationary phase. (**A and B**) Samples were taken at zero time, after 3 h of carbon starvation of exponential phase cells (**A**) or after 48 h of growth (**B**). Samples were then subjected to crosslinking at the indicated formaldehyde concentrations as described in “Materials and Methods.” The size of RNase PH was then determined by Western blot analysis using anti-FLAG-tag antibody. A band was present at approximately 84 kDa in the zero-time samples in both panels but was absent after 3 h of starvation or 48 h of growth. The molecular mass markers on the right side of each gel image provide a reference for the size of the RNase PH complex. (**C and D**) Formaldehyde crosslinking was carried out as described above using 0.2% formaldehyde. Following preparation of extracts by sonication, the samples were centrifuged to obtain a supernatant fraction and a cell pellet. As can be seen in panels C and D, the cross-linked complex resides largely in the supernatant fraction. In panel **C**, each fraction was treated with different ng amounts of RNase A. In panel **D**, only the supernatant fraction was treated using much higher µg amounts of RNase A. Experiments were repeated three times. RNase A amounts are indicated above each lane. The molecular mass markers in kDa are listed on the right side of each gel. The band at 84 kDa represents the RNase PH cross-linked complex. (**E**) Validation using Δ*rph* deletion strain. Wild-type (rph+) and Δrph deletion strains were subjected to formaldehyde crosslinking (0.5%) during stationary phase. Samples were collected at 0 and 1 h and analyzed by Western blot with anti-RNase PH antibodies. The 84 kDa band corresponding to the GroEL-RNase PH complex is present in wild-type cells at both time points but is completely absent in the Δ*rph* strain, demonstrating that RNase PH is required for formation of this complex.

As shown in Fig. 3, a higher molecular mass band of RNase PH at ~84 kDa was consistently observed in exponential phase samples. This band was generated at all formaldehyde concentrations tested, but was not present in the absence of formaldehyde, confirming that it is a crosslinked product generated by formaldehyde treatment. In contrast, the band was absent in cells starved for 3 h (panel A) or in stationary phase cells grown for 48 h (panel B). Likewise, the 84 kDa band was not generated upon formaldehyde treatment of stationary phase RNase PH-negative cells (panel E) indicating that its formation is dependent on the presence of RNase PH. Treatment with RNase A had no significant effect on the amount or size of the crosslinked band (panels C and D) suggesting that an RNA molecule was not a component of the crosslinked complex.

### Proteomic analysis reveals potential interaction of RNase PH with either of two proteins in exponential phase cells

To identify the protein crosslinked to RNase PH, LC-MS analysis of the formaldehyde crosslinked complex was carried out. Thirty-nine proteins organized into 32 distinct clusters were identified. Each cluster was characterized by its unique proteins and respective spectrum counts, indicating the level of detection confidence. The probability legend revealed high confidence levels for certain clusters with those in the green range (>95%) signifying robust identification based on the spectral data (Tables S1 and S2). The analysis identified two proteins as potentially being part of the formaldehyde cross-linked complex with RNase PH. One, the 57 kDa *E. coli* chaperonin, GroEL, had the highest unique spectra and unique peptide counts (unique spectra, 32, and unique peptides, 27), as shown in Table 1. Second was the 64 kDa protein phosphoenolpyruvate protein phosphotransferase (Pts1) with 20 unique spectra and 17 unique peptides (Table 1). RNase PH was detected as a 24 kDa protein with 19 unique spectra and 11 unique peptides (Table 1). Adding the mass of RNase PH to that of either GroEL or Pts1 gave a combined mass of 81 kDa or 87 kDa, respectively, in close approximation to the mass of the formaldehyde cross-linked complex of RNase PH at 84 kDa, determined by gel electrophoresis (Fig. 3).

**TABLE 1:** Listed are the various proteins identified by LC-MS in the formaldehyde crosslinked complex of RNase PH, along with their unique spectral counts and unique peptide counts^*a*^

Protein name and symbol	Unique spectral count	Unique peptide count	Molecular weight (kDa)	Molecular mass of protein in complex with RNase PH
Chaperonin protein (GroEL)	32	27	57.3	82.6
Phosphoenolpyruvate-protein phosphotransferase (Ptsl)	20	17	64.0	89.3
Ribonuclease PH (RNase PH)	19	11	25.3^*b*^	50.6
ATP synthase subunit beta (atpD)	18	10	50.3	75.6
Uncharacterized protein YeaG	17	14	74.4	99.7
Elongation factor (TufA)	13	10	43.4	68.7
Thioredoxin (trxA)	13	10	11.8	37.1
Succinate dehydrogenase flavoprotein subunit (sdhA)	13	10	65.1	90.4

^*a*^ Each protein was identified with a detection confidence level exceeding 95%. The proteins are identified by their common names and symbols and are presented with their molecular masses.

^*b*^ Full-length, active RNase PH has a molecular weight of 25.3 kDa. A shorter 24 kDa, largely inactive, form is found in many *E. coli* strains.

### Confirmation of GroEL in the cross-linked complex with RNase PH

**Fig 4:**
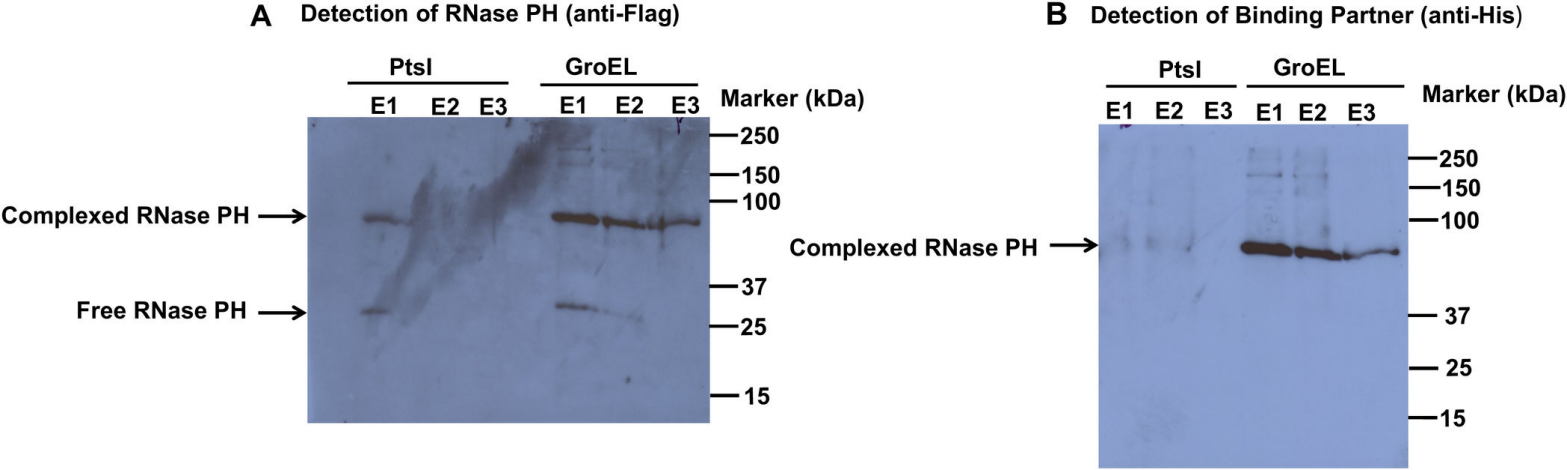
Identification of GroEL in the formaldehyde crosslinked complex with RNase PH. (**A**) Detection of RNase PH in crosslinked complexes. Wild-type cells expressing FLAG-tagged RNase PH were co-transformed with plasmids expressing either His-tagged PtsI or His-tagged GroEL. Cells were subjected to formaldehyde crosslinking, and FLAG-RNase PH was immunoprecipitated. Equal amounts of total protein from the initial cell lysates were used for each immunoprecipitation to ensure comparability. E1, E2, and E3 refer to elutions of the immunoprecipitated proteins which were eluted by boiling the protein-bound A/G agarose beads in SDS sample buffer for 5 min. Complexed and free RNase PH were detected by Western blot using anti-FLAG antibodies. RNase PH shows complex formation primarily with GroEL, with minimal interaction with PtsI. (**B**) Detection of binding partners in crosslinked complexes. The same samples from panel A were probed with anti-His antibodies to detect the His-tagged binding partners (PtsI or GroEL). The 84 kDa complex band containing RNase PH is detected specifically in GroEL-expressing samples, confirming GroEL as the primary binding partner.

To distinguish which of the two potential candidates identified by mass spectral analysis was present in the formaldehyde-crosslinked complex with RNase PH, strains were constructed that contained Flag-tagged RNase PH together with plasmids carrying either *groEL* or *pts1*, each with a His-tag. Each of these cells was subjected to the starvation and formaldehyde crosslinking protocol described above, immunoprecipitated and analyzed by Western blotting using anti-Flag or anti-His antibodies (Fig. 4). As shown in Panel A, RNase PH was detected in a relatively faint 84 kDa-band in the strain carrying the Pts1 plasmid (likely due to endogenous GroEL) and was present as a strong band in the strain with the GroEL plasmid. A band corresponding to non-crosslinked RNase PH at 28 kDa was also observed in each case. Most importantly, using anti-His antibody (Panel B), only GroEL, but not Pts1, was found to be present in the 84 kDa complex. Based on these data, we conclude that GroEL is the protein present in the 84 kDa formaldehyde crosslinked complex together with RNase PH.

### GroEL stabilizes RNase PH during starvation and stationary phase

**Fig 5:**
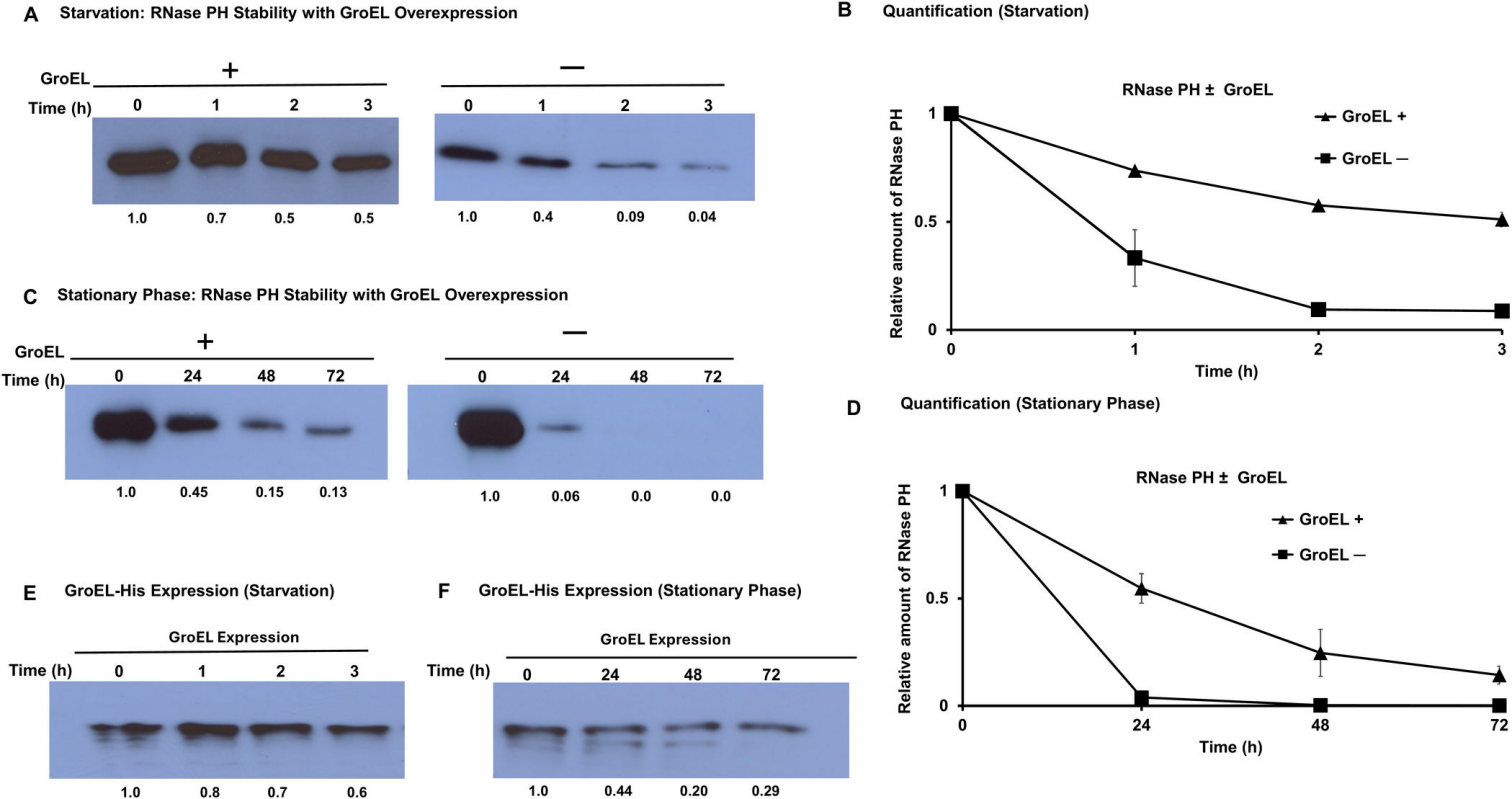
GroEL overexpression stabilizes RNase PH during nutrient deprivation. (**A**) RNase PH stability during starvation with GroEL overexpression. Cells containing GroEL expression plasmid (GroEL+) or empty vector control (GroEL−) were subjected to glucose starvation. Samples were collected at 0, 1, 2, and 3 h and analyzed by Western blot with anti-RNase PH antibodies. Numbers below lanes represent relative RNase PH levels quantified using ImageJ and normalized to *T* = 0. Values represent mean ± SEM from *n* = 3 biological replicates. (**B**) Quantification of data in panel A. Data represent mean ± SEM from three independent experiments. (**C**) Cells were grown to stationary phase and samples collected as 0, 24, 48, and 72 h. Western blot analysis performed as in panel A. Band intensities were quantified using ImageJ software and normalized to *T* = 0. Values represent mean ± SEM from *n* = 3 biological replicates. (**D**) Quantification of panel C. Data represent mean ± SEM from three biological replicates. (**E**) GroEL-His expression during starvation. Duplicate samples from panel A were probed with anti-His antibodies to detect plasmid-encoded GroEL-His expression. Samples were collected at 0, 1, 2, and 3 h of starvation. Numbers below lanes show relative GroEL-His levels quantified using ImageJ and normalized to *T* = 0. (**F**) GroEL-His expression during stationary phase. Duplicate samples from panel C were probed with anti-His antibodies to detect GroEL-His expression. Samples were collected at 0, 24, 48, and 72 h of stationary phase. Numbers below lanes show relative GroEL-His levels.

The data presented imply that GroEL forms a complex with RNase PH during exponential phase growth, but that this complex no longer is present in starved cells or in cells during stationary phase. Inasmuch as RNase PH is relatively stable during exponential phase but becomes unstable during starvation and stationary phase, these findings raise the possibility that GroEL serves to protect RNase PH during normal growth conditions but fails to do so under conditions of nutrient deprivation. If this conclusion is correct, then the presence of an increased amount of GroEL might stabilize RNase PH and prevent its degradation even during stationary phase and starvation. To test this idea, we increased the amount of GroEL protein using a *groEL* expression plasmid and assessed its effect on the stability of RNase PH during starvation and stationary phase. The data in Fig. 5 show that the stability of RNase PH was, in fact, significantly enhanced during starvation in cells containing the GroEL expression plasmid (Panels A and B). Likewise, the presence of the GroEL expression plasmid greatly stabilized RNase PH as it entered and continued through 72 h of stationary phase (Panels C and D). As shown in Panels E and F using anti-His antibody to detect the expression of His-tagged GroEL, the expression of plasmid continued to function throughout these experiments. These findings support the conclusion that the interaction of GroEL with RNase PH protects it against proteolytic degradation and that the absence of this interaction under conditions of nutrient deprivation renders RNase PH much more susceptible to the action of Lon protease. Moreover, enhanced expression of GroEL helps to stabilize RNase PH even during the stress conditions.

### RNase II activity is required for cell viability during stationary phase and for RNase PH degradation

A second protein that influences the degradation of RNase PH during nutrient deprivation is the 3′ to 5′ exoribonuclease RNase II. In earlier work from our laboratory (21), it was shown that cells devoid of RNase II lost viability during stationary phase and upon induced starvation, and the usual degradation of RNase PH was much less pronounced. However, those studies were carried out using an RNase II deletion strain, making it unclear whether the effects on viability and RNase PH were due to the absence of RNase II protein or the absence of RNase II activity.

**Fig 6:**
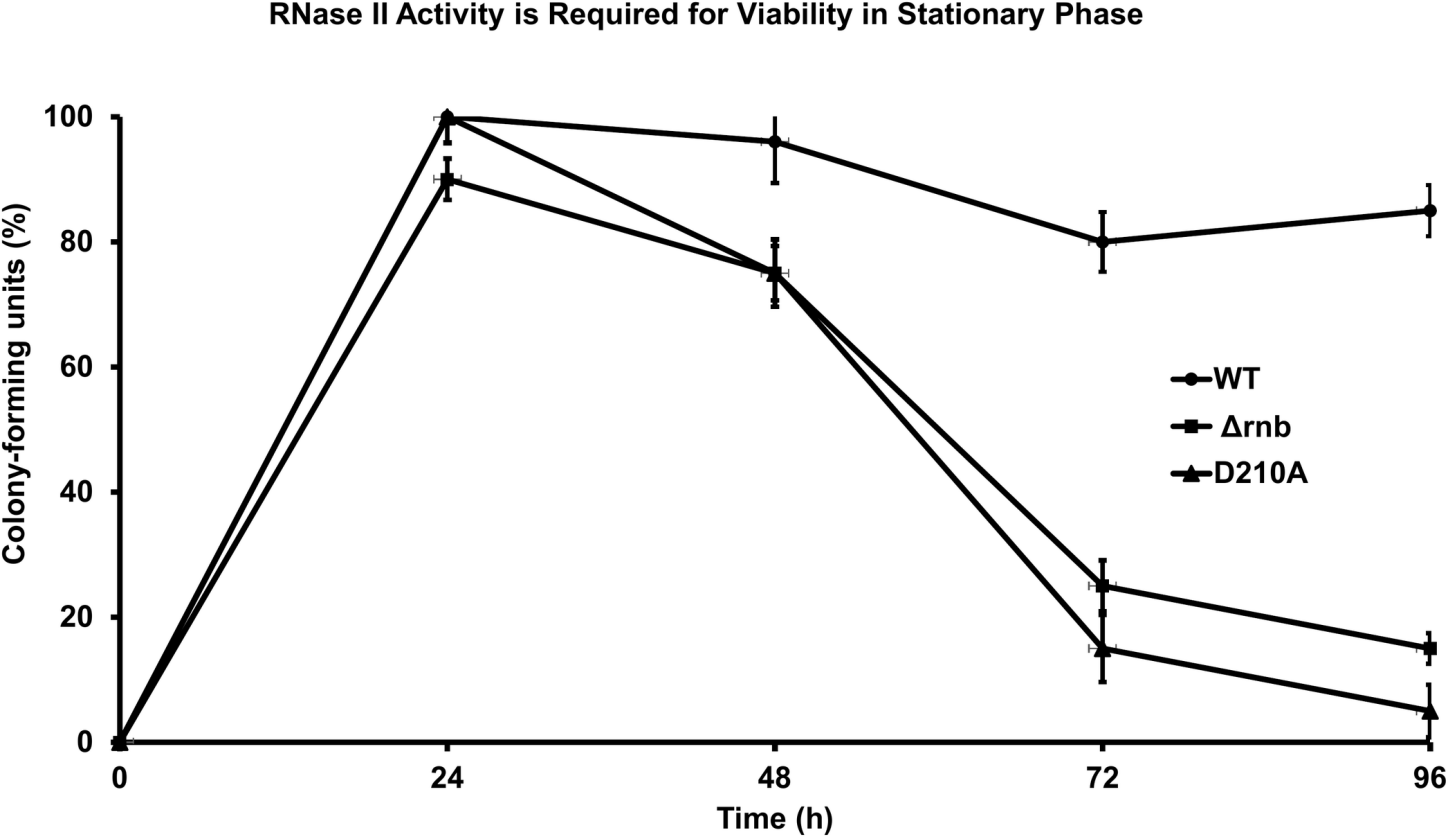
RNase II activity is required for bacterial viability during stationary phase. Cells were grown to stationary phase and samples were taken at 0, 24, 48, 72, and 96 h. Serial dilutions were plated on LB agar and colonies counted after overnight incubation at 37°C. Colony-forming units (CFU) of wild-type, Δrnb (RNase II deletion), and D210A (catalytically inactive RNase II mutant) are shown. Values are expressed as the percentage of CFU relative to *T* = 0 h. Data represent the mean ± SEM from *n* = 3 independent experiments.

**Fig 7:**
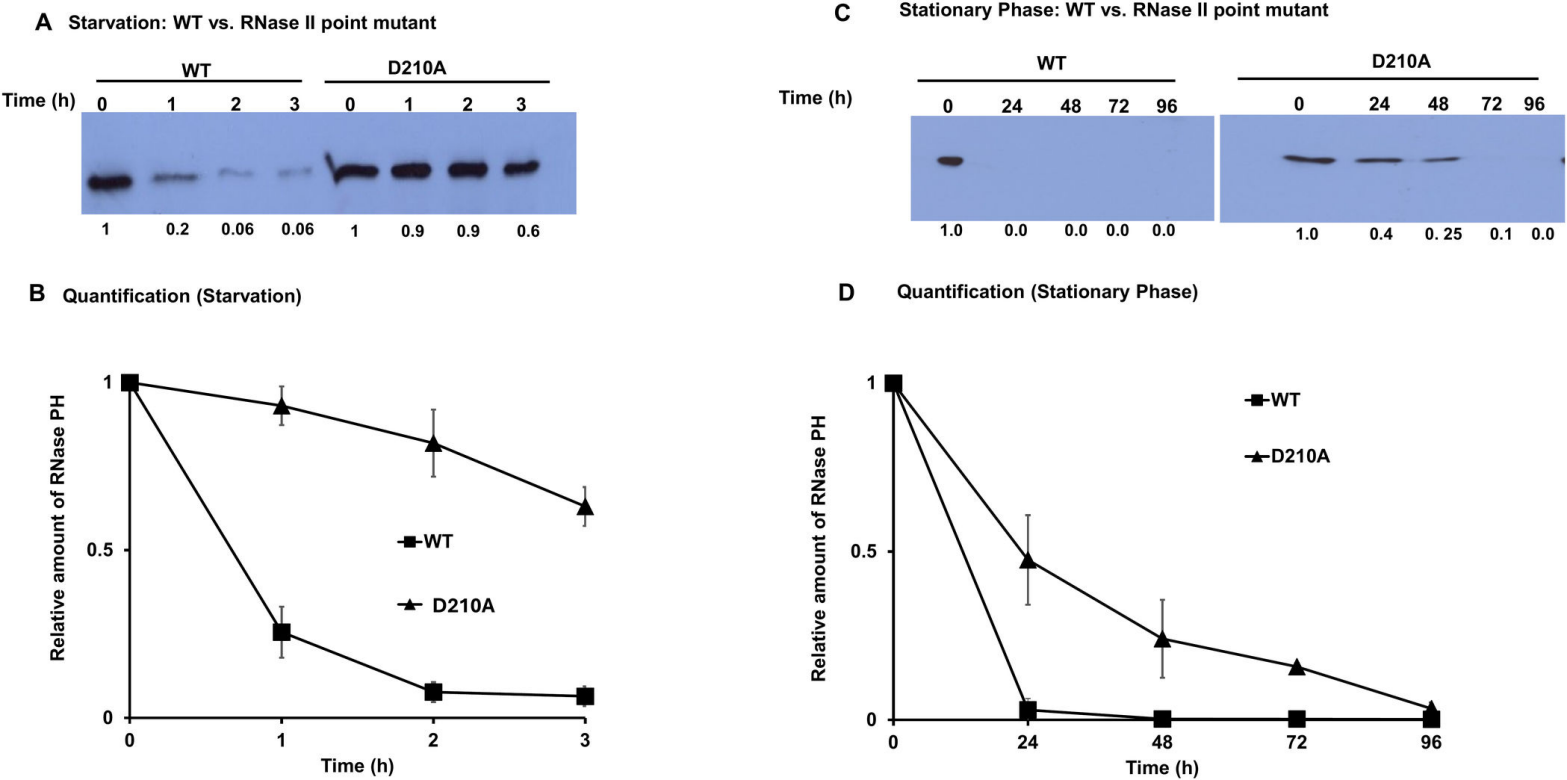
Stabilization of RNase PH in an RNase II point mutant (D210A) strain. (**A**) Wild-type and D210A (catalytically inactive RNase II) strains were subjected to carbon starvation and samples collected at 0, 1, 2, and 3 h. Western blot analysis was carried out with anti-RNase PH antibodies, as described. Numbers below lanes represent the relative RNase PH levels quantified using ImageJ and normalized to *T* = 0. Values represent mean ± SEM from *n* = 3 biological replicates. (**B**) Quantification of data in panel** A**. (**C**) Cells were grown to stationary phase and samples collected at 0, 24, 48, 72, and 96 h. Western blot analysis was carried out as in panel **A**. Band intensities were quantified using ImageJ, normalized to *T* = 0. Values represent mean ± SEM from *n* = 3 biological replicates. (**D**) Quantification of data in panel **C**.

To resolve this outstanding question, a strain was constructed that contained a point mutation in the *rnb* gene resulting in a loss of RNase II activity, but which retained RNase II protein. This strain was compared to the previously studied RNase II deletion strain for cell viability and RNase PH stability. As shown in Fig. 6, the strain lacking RNase II activity lost viability in stationary phase in the same manner as the RNase II deletion strain. Thus, cell numbers were reduced by ~25% after 48 h and by over 80% after 96 h for both strains. Likewise, in the absence of RNase II activity, RNase PH was greatly stabilized during both starvation (Fig. 7, panel A) and during stationary phase (Fig. 7, panel C). These findings clearly indicate that the effects of RNase II removal are due to the absence of its activity and are not a consequence of simply removing RNase II protein (21). However, it is not yet understood how the absence of RNase II activity stabilizes RNase PH during nutrient deprivation.

## DISCUSSION

The studies presented here provide us with a much deeper understanding of the regulation of RNase PH that occurs under conditions of nutrient deprivation. Previously, all that was known was that the level of RNase PH decreased as much as 90% during 3 h of carbon starvation and that the decrease did not occur in cells lacking RNase II (21). However, nothing was known about the mechanism of RNase PH removal or the factors that might be involved in the process. Based on our new findings, it is now clear that in addition to starvation, RNase PH levels also decrease dramatically during extended stationary phase, and that the loss of RNase PH both in stationary phase and during carbon starvation is due to proteolysis, primarily by the protease Lon. While our data clearly identify Lon as the principal protease responsible for RNase PH degradation, the results from the double protease mutant strains suggest that other proteases may also contribute to some extent. However, it is also possible that the simultaneous loss of two major proteases triggers an enhanced cellular stress response leading to the upregulation or activation of other proteolytic pathways that can also target RNase PH.

Moreover, based on our findings, it is now apparent that RNase PH forms a complex with the chaperonin GroEL in growing cells, whereas this complex is not detectable in stationary phase cells or during starvation. These data imply that the interaction with GroEL stabilizes RNase PH during the growth phase, but in the absence of complex formation in stationary phase or during starvation, it becomes susceptible to proteolysis. This conclusion was strengthened by the observation that the overexpression of GroEL helps to stabilize RNase PH even under conditions of nutrient deprivation. Given that RNase PH is functionally active during growth, we might expect its interaction with GroEL to be transient and dynamic, and this was supported by the predicted structure of the RNase PH-GroEL complex determined by AlphaFold analysis which indicated a weak modeling score and low confidence for the binding interface (data not shown).

RNase PH is part of a growing list of ribonucleases that are dramatically regulated in response to physiological conditions (8). In this case, RNase PH levels decrease as much as 90% under conditions of nutrient deprivation such as occur during carbon starvation or extended stationary phase. Previous work had shown that ribosomes begin to undergo degradation under these conditions due to reduced translation and a resultant increase in the free ribosome subunit population that is much more susceptible to attack by RNases (11). RNase PH is necessary to initiate the degradation of 30S ribosome subunits and thereby helps to provide a source of nutrients under these stress conditions (14, 15). However, if left uncontrolled, the ribosome degradation can become so extensive that it affects cell viability due to a large accumulation of rRNA fragments (21). In wild-type cells, this problem is averted by lowering the amount of RNase PH present thereby reducing overall ribosome degradation. However, as was shown previously, in mutant cells lacking the exoribonuclease RNase II, RNase PH levels do not decrease, resulting in excessive ribosome degradation and ultimately to loss of cell viability (21). Thus, RNase PH is a central player in cell survival during stress conditions, and these findings explain why loss of the *rph* gene is one of the most frequently observed mutations in *E. coli* cells undergoing laboratory evolution under stress conditions (16–18). Consequently, understanding how RNase PH is regulated is of prime importance to understanding cell survival during stress.

Our finding that GroEL plays a major role in RNase PH stabilization and protection against proteolysis is somewhat surprising given that its primary role as a chaperonin is thought to be in facilitating protein folding and preventing interactions that could lead to protein aggregation (24). Interestingly, RNase PH does tend to aggregate (25), but it is not yet known how this property might be related to the interaction with GroEL reported here. It has been suggested that interaction with GroEL may directly promote the degradation of certain proteins (26, 27), but we are unaware of other examples in which interaction with GroEL protects against proteolytic degradation, as we have observed here for RNase PH in growing cells.

An important aspect of our proposed model is the relative cellular concentrations of GroEL and RNase PH. For the interaction with GroEL to substantially protect the majority of the RNase PH pool from degradation during exponential growth, it would be expected that GroEL is present in stoichiometric excess over RNase PH. In fact, quantitative proteomics studies have established that during exponential phase, *E. coli* cells contain approximately 8,000 to 10,000 GroEL monomers, whereas RNase PH is present at much lower levels, estimated at 200 to 300 molecules per cell (28). This represents a molar ratio of roughly 30:1 to 50:1. This substantial physiological excess of GroEL supports the conclusion that the chaperone capacity is sufficient to sequester and protect the RNase PH pool during growth.

This, of course, raises the question of why the interaction of RNase PH with GroEL ceases in starved cells and in cells in stationary phase. The most straightforward explanation would be a reduction in the amount of GroEL available for interaction with RNase PH under these conditions, and some work supports this possibility. Thus, our findings are consistent with a report that *groEL* is downregulated in *E. coli* long-term stationary phase (LSP) cells in LB medium (22). Likewise, GroEL was found to decrease and then essentially disappear in *S. typhimurium* cells during stationary phase (29). On the other hand, genome-wide proteomic analyses have suggested that GroEL increases slightly in stationary phase cells (28, 30). Nevertheless, despite these contradictory findings, the fact that the overexpression of GroEL protects RNase PH against degradation strongly suggests that GroEL levels are normally insufficient for complete protection under the nutrient deprivation conditions. Clearly, further work will be necessary to determine whether a reduction in the amount of GroEL during stationary phase plays a role in the instability of RNase PH under such conditions.

It should be noted that although overexpression of GroEL protects against proteolysis in both starvation and stationary phase, the effect is more pronounced during the acute stress of starvation. It is not yet clear whether this is simply due to the much shorter time frame of the starvation experiment (3 h vs 72 h) or whether other regulatory processes that normally occurs during the gradual depletion of nutrients during stationary phase might be responsible. During this prolonged period, it is also possible that overexpressed GroEL is gradually depleted or that other compensatory regulatory pathways are activated. In addition, under both physiological conditions, the protection by GroEL is incomplete. One possibility that might explain this finding is that under these stress conditions, there is insufficient GroEL given its greater need to protect other proteins. Furthermore, the functional GroEL chaperonin machine requires the cofactor GroES for full activity, which was not co-overexpressed in our experiments. It is conceivable that over a prolonged 72-h period, the endogenous supply of GroES becomes the limiting factor for the protective function of the excess GroEL. This cofactor limitation may explain why the protection is incomplete and allows for the gradual degradation of RNase PH observed in stationary phase even when GroEL is abundant.

Since the stability of RNase PH appears to be significantly influenced by the molecular chaperone, GroEL, regulation of GroEL itself becomes of critical importance when considering its impact on RNase PH. The expression of GroEL is known to be sensitive to various cellular stresses, including heat shock, carbon source deprivation, and the accumulation of abnormal proteins (31–33). The observed downregulation of *groEL* in *E. coli* long-term stationary phase cells (22) would be consistent with our findings in which RNase PH stability decreases over extended periods in stationary phase and would support a model whereby cellular conditions leading to reduced GroEL levels could indirectly render RNase PH more susceptible to proteolytic degradation by enzymes like Lon, which we have identified as a key player in RNase PH turnover during stationary phase and starvation.

These considerations raise the question of how RNase II fits into the picture. As shown earlier (21), RNase II has a direct impact on RNase PH levels during nutrient deprivation. In the presence of RNase II, RNase PH levels decrease dramatically, whereas in an RNase II mutant strain, RNase PH levels remain unchanged leading to loss of viability in stationary phase. The important finding, presented here, that RNase II activity is required for its physiological effects, strongly suggests the involvement of an RNA molecule in the regulation of RNase PH. One possibility, not explored in the current work, could involve RNase II indirectly affecting RNase PH stability by modulating the expression or availability of GroEL. For instance, RNase II could influence the turnover of *groEL* mRNA or transcripts of other regulators that, in turn, affect GroEL protein levels. Previous genome-wide analyses have, indeed, suggested that *groEL* transcript levels can be altered in *rnb* mutants (34) although the direction and magnitude of this effect might depend on specific growth conditions (35). It is also known that a small RNA, CpxQ, interacts with GroEL mRNA (36), and conceivably, RNase II could affect levels of this molecule and thereby influence expression of GroEL. Further studies of this important aspect of RNase PH regulation will be required to unravel the role of RNase II.

Our findings also reveal an interesting nuance regarding the role of RNase II in regulating RNase PH stability under different stress conditions. While the absence of RNase II activity leads to a dramatic and near-complete stabilization of RNase PH during the 3-h acute starvation period, the effect is less absolute during the prolonged stress of stationary phase. In the stationary phase, RNase PH is only partially stabilized in the RNase II mutant, with its levels still declining significantly over 96 h, albeit more slowly than in the wild-type strain. This difference likely reflects the distinct cellular responses to acute vs chronic nutrient deprivation. Similar to the incomplete protection afforded by GroEL overexpression over extended periods, the partial degradation of RNase PH in the RNase II mutant during stationary phase may suggest the activation of slower, alternative regulatory or degradation pathways that are not prominent during short-term stress.

In conclusion, this study underscores the pivotal role of the chaperone GroEL in regulating RNase PH stability. The dynamic regulation of GroEL expression in response to the cell’s physiological state, especially during prolonged stress and stationary phase, may be a key determinant of RNase PH’s fate. While RNase II action undoubtedly plays a role in this regulatory network, our current findings highlight that GroEL-mediated protection, and factors governing GroEL availability, are central to understanding RNase PH regulation. Future investigations will be necessary to dissect the specific mechanisms by which GroEL expression and activity are modulated under different stress conditions relevant to RNase PH function and how these connect with the proteolytic machinery of the cell.

## MATERIALS AND METHODS

### Materials

Oligonucleotide primers were synthesized and purified by Sigma Genosys. Taq DNA polymerase was purchased from New England Biolabs, Inc. Goat anti-mouse IgG-HRP was obtained from Santa Cruz Biotechnology, Inc. Monoclonal anti-Flag M2 antibody produced in mouse and all other reagent grade chemicals were obtained from Sigma-Aldrich.

### Bacterial strains

*E. coli* MG1655^∗^ (seq) I^−^, which is RNase PH^+^, was considered to be the wild type for this study; MG1655 I^−^ is its RNase PH^−^ counterpart (14). This latter strain served as the recipient for the RNase II mutation, which was introduced via P1 transduction (14, 37). The RNase II mutated strain was that used in the study of Sulthana et al. (21). DNA encoding the 2×Flag sequence was fused to the N terminus of the chromosomal *rph* gene following a previously published recombineering protocol (38) as detailed in the protocol from Sulthana et al. (21). Antibiotics, when present, were at the following concentrations: kanamycin, 50 µg/mL ampicillin, 100 µg/mL; chloramphenicol, 34 µg/mL.

### Generation of MG1655* RNase II D210A mutant strain

A point mutation was introduced at the active site of RNase II to disrupt its enzymatic activity, allowing us to specifically study the role of RNase II’s enzymatic function in the regulation of RNase PH. Recombineering was performed using a single-strand oligonucleotide, RNase II D210-A, as described by Costantino and Court (39), to create an RNase II (D210A) mutation in the *rnb* gene in *E. coli* MG1655*.

A single-stranded DNA (ssDNA) oligonucleotide, 70 nucleotides in length, was designed that carried the desired point mutation GAC to GCG at its center (5′TTTGTCATCCGGCAACGCCTTAGCGAAAAGGGCCGCATCCATATCTTCTGTGCTGGCACTGTCGATGGTG3′). Cells were grown to mid-log phase and made electrocompetent by washing with ice-cold water followed by washing with a 10% glycerol solution. ssDNA oligonucleotides were mixed with the electrocompetent cells and electroporated following a standard protocol. Immediately after electroporation, super optimal broth catabolite repression (SOC) medium was added to the cells to aid their recovery. The cells were then incubated at 37°C for 1 h. Following recovery, cells were plated on selective agar plates and incubated overnight at 37°C. Individual colonies were selected, and the presence of the desired mutation was confirmed by DNA sequencing.

#### *rph* deletion strain

The Δ*rph* strain (ΔRNase PH) used for the validation experiments shown in Fig. 3E was constructed by the deletion of the *rph* gene encoding RNase PH. This strain lacks RNase PH protein as confirmed by Western blot analysis (data not shown). The Δ*rph* strain was grown under conditions identical to the wild-type strain for crosslinking experiments.

### Growth conditions for carbon starvation experiments

A single colony was inoculated into 3 mL of M9/0.2% glucose medium and shaken overnight at 31°C. The overnight culture was diluted 1:100 into 30 mL of M9/0.2% glucose medium and shaken at 31°C to A_550_ of 0.2–0.3. Cells were collected by centrifugation at 7,000 rpm at room temperature for 20 min. After removal of the supernatant fluid, cells were washed three times with 30 mL of M9 medium and resuspended in 30 mL of M9 medium at 42°C. At regular time intervals, cells were removed, tested for viability in triplicate at 37°C, and used for measurement of RNase PH.

### Growth conditions for stationary phase experiments

A single colony was inoculated into 5 mL of M9/0.2% glucose medium and shaken overnight at 31°C. The overnight culture was diluted 1:100 into 50 mL of M9/0.2% medium and grown at 42°C. Samples were removed every 24 h, tested for viability in triplicate at 37°C, and used for measurement of RNase PH.

### Measurement of RNase PH by Western blot during carbon starvation and stationary phase

Western blot analysis was used to measure RNase PH levels under various growth conditions. Samples were collected as described in the growth experiments. Twenty milliliters of culture from each time point was centrifuged and resuspended in 0.4 mL of solution containing 20 mM Tris-Cl, pH 7.5, 1 mM dithiothreitol, 500 mM KCl, and 0.1 mM phenylmethylsulfonyl fluoride. Extracts were prepared by sonication on ice using three 20 s pulses with 20 s intervals, followed by centrifugation at 15,000 rpm for 20 min to remove cell debris. Protein concentration of the extracts was determined by the method of Bradford using bovine serum albumin as the standard.

Protein samples (10 µg/well) were resolved by 8% SDS-PAGE and transferred to a PVDF membrane by electroblotting. The membrane was treated with nonfat milk (5%) in TBST (100 mM Tris-HCl, 150 mM NaCl, 0.5% Tween 20 at pH 7.5) at room temperature for 2 h. The membrane was washed 3× with TBST for 10 min each. The membrane was then treated overnight at 4°C with a 1:2,000 dilution of anti-FLAG tag serum in TBST containing 0.5% nonfat milk. Anti-mouse IgG HRP conjugate was used as the secondary antibody (1:5,000 dilution), and the detection was with *p*-coumaric acid and Luminol. Underexposed films were used for quantitation.

#### Quantification of Western blot bands

Western blot band intensities were quantified using ImageJ software (National Institutes of Health, https://imagej.net/ij/). For each blot, rectangular regions of interest (ROIs) of identical dimensions were drawn around individual bands. Mean gray values were measured for each band and for background regions. Background-corrected intensities were calculated by subtracting the background value from each band value. Relative band intensities were then normalized to the *T* = 0 time point (set to 1.0) for each experiment. All quantifications represent mean ± standard error of the mean (SEM) from at least *n* = 3 independent biological replicates unless otherwise noted. Statistical analysis was performed using Student’s *t*-test where indicated.

### *In vivo* cross-linking of RNase PH and protein analysis

Formaldehyde cross-linking experiments were conducted to ascertain whether RNase PH interacted with any proteins or RNAs under the different growth conditions. WT cells containing the 2× Flag *rph* gene were grown in M9/glucose at 31°C to early exponential phase (*A*_550_ = 0.3–0.4) and then starved for glucose for 3 h at 42°C in M9 medium. Formaldehyde (37%, wt/wt) was added to the zero time and 3-h glucose-starved samples to a final concentration varying from 0.05% to 2%, and samples were incubated for 20 min at 31°C. The reaction was terminated by the addition of glycine to a final concentration of 125 mM and incubating for 5 min at room temperature. Cells were collected by centrifugation and resuspended in 150 µL of loading buffer. Portions of 20 µL were heated at either 60°C for 10 min or at 100°C for 20 min to reverse the cross-linking. Samples were then loaded on a polyacrylamide gel, fractionated by SDS-PAGE, and analyzed by Western immunoblotting using anti-Flag monoclonal antibody. Experiments were repeated three times. Similar experiments were also performed in stationary phase cells. WT cells containing the 2× Flag *rph* gene were first grown in M9/glucose medium at 31°C to an *A*_550_ of 0.4–0.5 and then transferred to 42°C for the additional growth until 48 h. Samples from the exponential phase at 31°C and after 48 h of stationary phase growth at 42°C were subjected to formaldehyde cross-linking as described above.

### Immunoprecipitation and LC-MS analysis of the cross-linked RNase PH complexes

After cross-linking and termination of the reaction as described above, cells were harvested and lysed in Immunoprecipitation (IP) lysis buffer (50 mM Tris-HCl, pH 7.4, 150 mM NaCl, 1 mM EDTA, 1% Triton X-100) supplemented with protease inhibitors (Complete, Mini Protease Inhibitor Cocktail, One Tablet/10 mL solution). The lysates were centrifuged at 14,000 × *g* for 15 min at 4°C to remove cell debris and then incubated for 2 h with protein A/G agarose beads to eliminate nonspecific binding. An isogenic control antibody was used as a negative control to help differentiate specific antibody binding from nonspecific interactions. This control antibody matched the species, isotype, and subclass of the primary antibody but does not target any antigen in the sample. The supernatant fluid was incubated with anti-RNase PH antibody overnight at 4°C with gentle rotation. Protein A/G agarose beads were added to the lysate and incubated for 2 h at 4°C. The beads were washed three times with IP lysis buffer and once with PBS, and the immunoprecipitated proteins were eluted by boiling the beads in SDS sample buffer for 5 min. The cross-linked proteins were then subjected to SDS-PAGE and visualized by Coomassie staining. For LC-MS analysis, the cross-linked protein band corresponding to approximately 84 kDa was excised from the gel and sent to the Proteomics Core at Scripps Research Institute for analysis. The Scaffold file obtained was examined using the Scaffold program VIEWER. Peptide identifications obtained from the LC-MS/MS data were cross-referenced with both the *E. coli* protein FastA database and the UniProt database. Filters were applied to only display proteins identified based on a minimum of five peptides and a false discovery rate (FDR) of 1% or less. Results are presented as the total number with their unique spectral counts and unique peptide counts. Each protein was identified with a detection confidence level exceeding 95%. The proteins are listed with their common names, symbols, and molecular masses in kDa.

### Validation of interaction between RNase PH and GroEL by immunoprecipitation

To confirm the interaction between RNase PH and GroEL, *E. coli* MG1655^∗^(seq) I^−^ cells containing an N-terminal Flag-tagged RNase PH gene were transformed with plasmids carrying *GroEL* and *PtsI* that expressed their respective proteins with His-tags. Cells were grown to mid-log phase under conditions as described above for the stationary phase and starvation experiments. For electroporation of the plasmids, cells were made electrocompetent by washing with ice-cold water followed by a 10% glycerol solution. Plasmids were mixed with the electrocompetent cells and subjected to a standard electroporation protocol. Post-electroporation, LB broth was added to aid cell recovery, and the cells were incubated at 37°C for 1 h. After recovery, cells were plated on selective antibiotic plates and incubated overnight at 37°C to select strains containing the *GroEL* and *PtsI* plasmids. *In vivo* cross-linking was carried out on these cells at 60°C for 10 min using 0.01% formaldehyde under stationary phase and starvation growth conditions. Following cross-linking, cell lysates were incubated with an anti-Flag antibody to target the tagged RNase PH. The resulting antibody-protein complexes were captured using protein A/G agarose beads. After washing the beads to remove non-specifically bound proteins, the captured complexes were eluted in three sequential fractions (E1, E2, and E3) by boiling the beads in SDS sample buffer for 5 min. Cross-linked proteins were then subjected to SDS-PAGE for separation and subsequent analysis. RNase PH was detected using anti-Flag antibody, while GroEL and PtsI were detected using anti-His antibody.

## SUPPLEMENTAL MATERIAL

Supplemental material mbio.00443-26-s0001.docx Fig. S1; Tables S1 to S3.

ASM does not own the copyrights to Supplemental Material that may be linked to, or accessed through, an article. The authors have granted ASM a non-exclusive, world-wide license to publish the Supplemental Material files. Please contact the corresponding author directly for reuse.
