# Supplementary material for: Regulation of RNase PH during nutrient deprivation: the role of proteases, GroEL, and RNase II
Source: mBio. 2026 Apr 7;17(5):e00443-26. doi: 10.1128/mbio.00443-26 (PMC13170331; doi:10.1128/mbio.00443-26)

**SUPPLEMENTARY RESULTS**

**Differential stability of RNase PH in exponential phase and stationary phase cells is not due to acetylation**

The aim of this experiment was to determine whether RNase PH was acetylated and whether it varied under any physiological conditions.


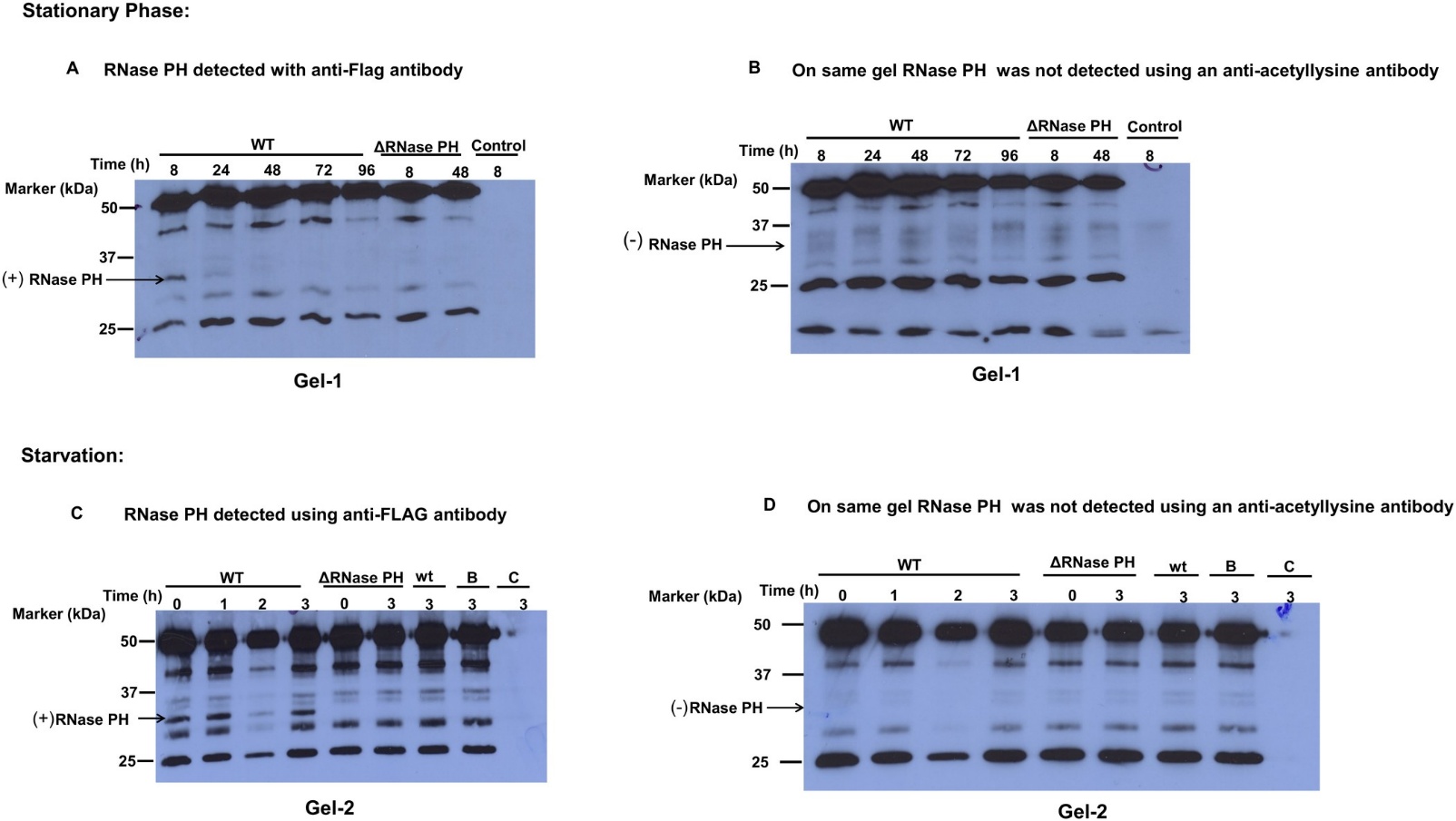


**Figure S1: Analysis of RNase PH Acetylation under Various Growth Conditions**

Acetylation of RNase PH was assessed during stationary phase and starvation at different time points using an anti-acetyllysine antibody. FLAG- tagged RNase PH was pulled down from cell extracts using anti-FLAG antibody and Protein G agarose beads and samples were run on 8% SDS-PAGE. Acetyllysine was detected with anti-acetyl-lysine antibody and RNase PH was detected using anti-FLAG antibody. Shown are representative gels from experiments carried out at least twice. Panels A and B. RNase PH detected with anti-Flag antibody (Panel A) or same gel using an anti-acetyllysine antibody (Panel B). Similar analysis was done for starved cells. Panels C) and D. RNase PH detected using anti-FLAG antibody (Panel C) or same gel but using an anti-acetyllysine antibody (Panel D). (+) indicates presence of FLAG- tagged RNase PH; (─) minus indicates absence of band of acetylated RNase PH. Lane wt, without FLAG-RNase PH; lane B, only anti-FLAG antibody without lysate; lane Control, lysate and Protein G.

The activity of anti-acetyllysine antibody was confirmed on a separate gel using RNase II as a substrate (data not shown).

**Spectral and peptide counts from LC-MS analysis of the RNase PH cross-linked complex**

Proteins identified in the formaldehyde cross-linked complex of RNase PH, along with their unique spectral (Table S1) and peptide counts (Table S2) are shown. They are identified by their common names and symbols, their molecular weight and with a detection confidence level exceeding 95%.

**Table S1.** Total Unique Spectrum Counts from LC-MS Analysis of the RNase PH Cross-linked Complex

Proteins identified in the formaldehyde cross-linked complex of RNase PH are listed with their common names, symbols, and molecular weights. The table displays the total unique spectral counts for each protein, all identified with a detection confidence level exceeding 95%.


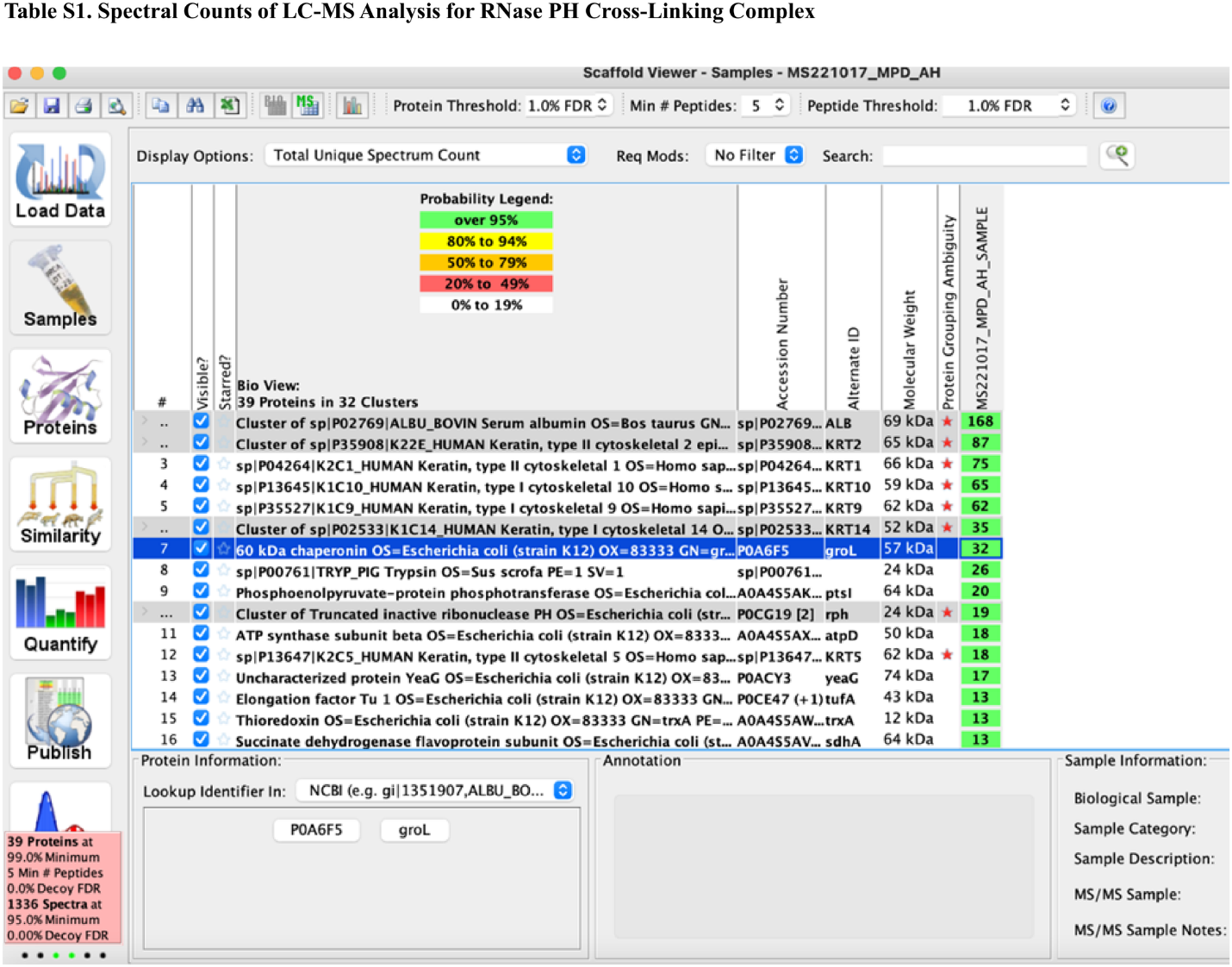


**Table S2.** Total Unique Peptide Counts from LC-MS Analysis of the RNase PH Cross-linked Complex

This table identifies proteins within the formaldehyde cross-linked complex of RNase PH based on unique peptide counts. Each protein is identified by common name, symbol, and molecular weight with a detection confidence level exceeding 95%.


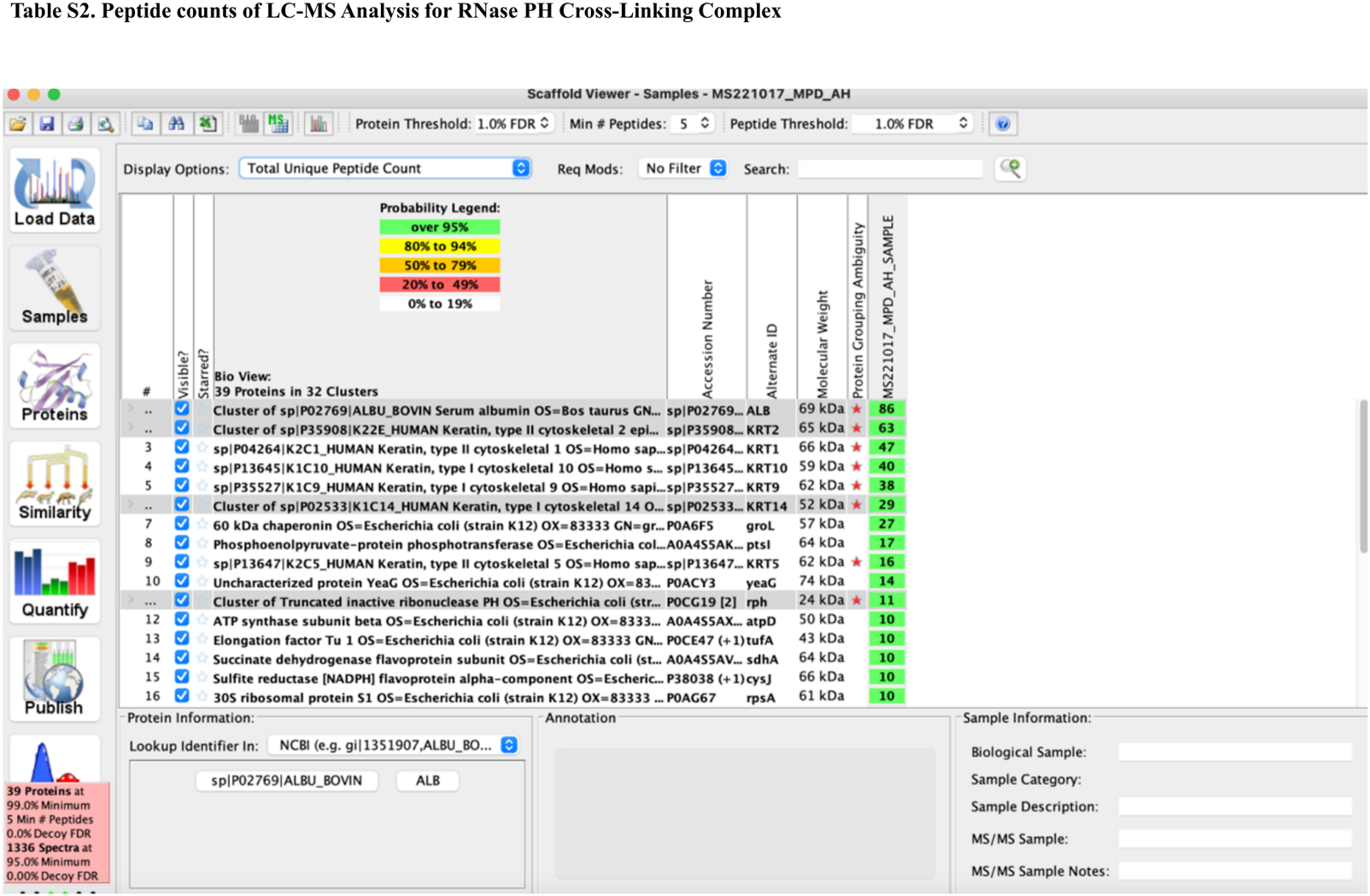


**Spectral and peptide counts from LC-MS analysis for GroEL within the RNase PH cross-linked complex**

The table shows the peptides identified in GroEL in the formaldehyde cross-linked complex of RNase PH, including their unique spectral counts and unique peptide counts, each identified with a detection confidence level exceeding 95%. The sequence coverage map highlighted in yellow, displayed significant coverage for the target protein (61%). This protein had 27 unique peptides and 32 unique spectra. The scores and coverage percentages indicate robust identification.

**Table S3.** Spectral and Peptide Analysis for GroEL within the RNase PH Cross-linked Complex

Detailed identification of GroEL (60 kDa chaperonin) peptides within the cross-linked complex. The protein was identified with 27 unique peptides and 32 unique spectra, achieving 61% sequence coverage (highlighted yellow in primary data). All peptides listed achieved a detection confidence level exceeding 95%


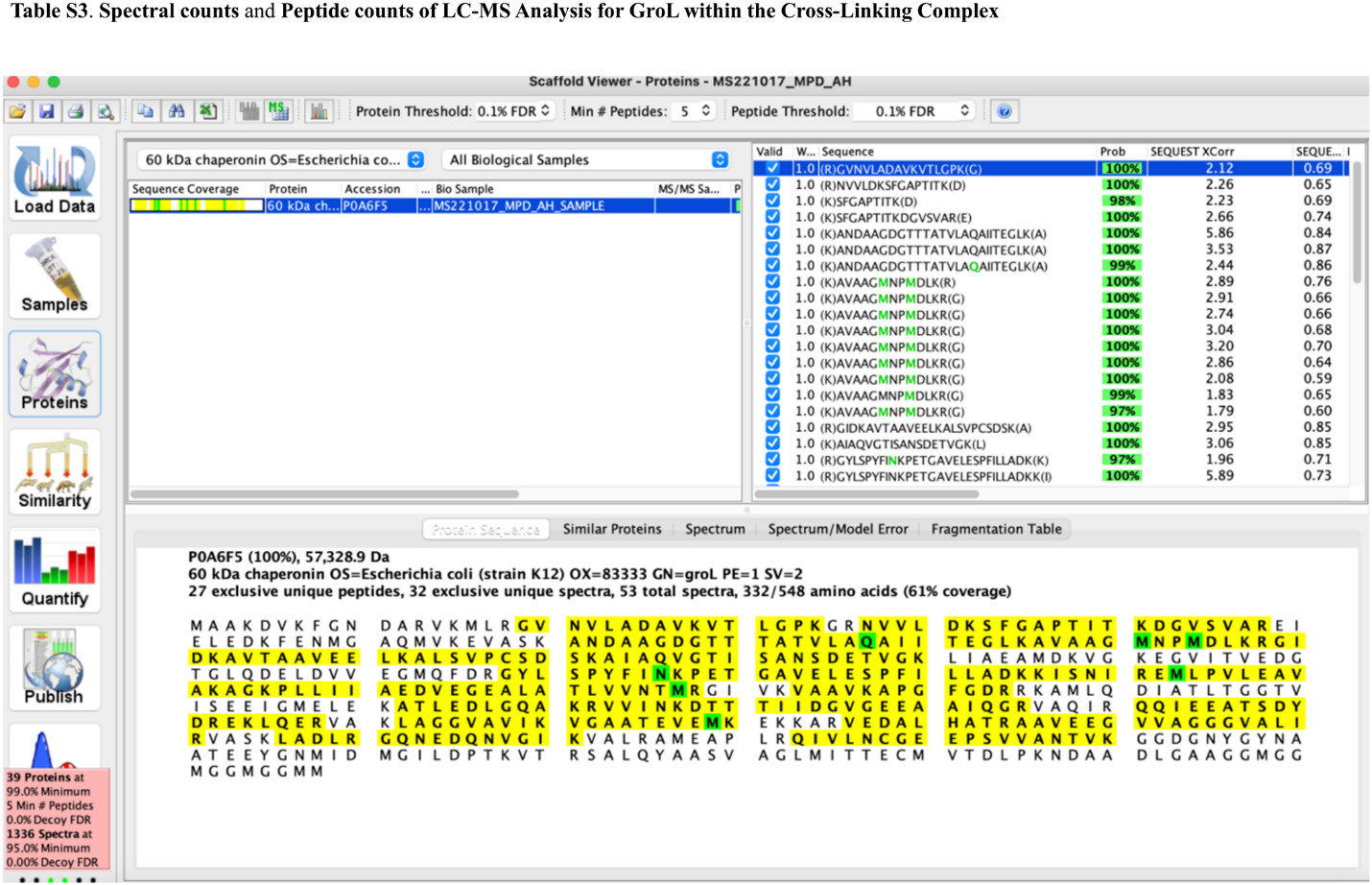

Supplement: Supplemental material — Fig. S1; Tables S1 to S3. [file mbio.00443-26-s0001.docx]
